# Blinded by Attachment: Examining the Overconfidence Bias of Sports Fans’ Intertemporal Ticket Purchase Decisions

**DOI:** 10.3390/bs13050405

**Published:** 2023-05-12

**Authors:** Wonsok Frank Jee, Moonsup Hyun

**Affiliations:** 1 School of Marketing, Entrepreneurship, Sport Management, Hospitality and Tourism Management, Western Carolina University, Cullowhee, NC 28723, USA; 2 Department of Business and Economics, Utica University, Utica, NY 13502, USA

**Keywords:** sport, involvement, overconfidence bias, inter-temporal choice, risk, uncertainty

## Abstract

Optimally deciding on the best deal for sport event tickets requires the ability to evaluate risk and make informed decisions in uncertain environments. This study examines how individual trait factors, such as experience, expertise, and involvement, influence consumers’ decision-making process when purchasing tickets online for sporting events. To examine and test the study hypotheses, 640 respondents from a Qualtrics survey panel were recruited from geographically confined subjects of New York City sports fans over a ten-day data collection period. The research subjects were surveyed to assess their perception of the expected likelihood of obtaining event tickets at a lower rate (ELR) and the expected likelihood that tickets would remain available (ETA) as the event day approached. MANOVA showed that there was a significant effect of the time period on the participants’ ETA and ELR risk assessments [Λ = 0.954, *F* (18, 1262) = 1.653, *p* < 0.05]. The ETA was highest ten days before the event and lowest the day before the event, with a similar pattern observed for the ELR. The mediation path analysis showed that fan involvement had a strong positive correlation with confidence (*B* = 0.496, *p* < 0.001). Confidence, in turn, was a significant predictor of the ELR (*B* = 5.729, *p* < 0.05) but not for the ETA (*B* = 1.516, *p* = 0.504). The positive mediation of confidence between fan involvement and the ELR indicates that consumers with higher fan involvement tend to have overconfidence in their ability to evaluate the uncertain purchase environment, which ultimately impacts their risk perception and decision-making. The study highlights the importance of considering both temporal and psychological factors when assessing the likelihood of ticket purchases and provides behavioral insights for sports marketers and ticket distributors.

## 1. Introduction

The act of searching for the most favorable deal while buying tickets for sporting events over a period of time is fundamentally comparable to a behavior observed in behavioral ecology known as foraging and patch exploitation [1,2]. Optimization models have been utilized by behavioral ecologists to examine how animals seek out natural food resources at regular intervals while considering uncertain time and environmental constraints [3]. Similarly, consumers engage in information foraging and search to make predictions and decisions on when to purchase tickets based on various indicators and evaluations of uncertainty in the pre-sale ticketing environment [4].

Research in the field of sports has highlighted the significant level of uncertainty associated with pre-sale ticketing, which makes it inherently difficult to predict demand and prices for sports events [5]. This uncertainty is influenced not only by factors related to time and environmental variables (such as temperature, precipitation forecast, time of the event, part of season, weekday/weekend, days before event, etc.) [6], but also by dynamic team and individual performance factors (such as star player injuries, home and team winning percentage, season rankings, playoff contention, etc.) [7,8], which has been found to have a significant impact on game attendance and ticket prices. Sports fans value the unpredictability of the product, which can change rapidly and frequently throughout the season, and this ultimately affects their perception of the product’s usefulness [9,10].

Thus, in the context of pre-sale ticketing, the value of a sport ticket changes constantly, making it challenging for consumers to accurately determine the optimal time to purchase tickets for a sporting event [8]. In a perfect market with complete information, consumers would have access to all the relevant information necessary to make informed decisions about when to purchase sports event tickets [11]. Nevertheless, due to time and resource constraints, as well as inherent cognitive limitations, consumers often rely on quick and automatic decision-making rules that are influenced by decision heuristics based on intuition [12]. For example, experienced consumers in the pre-sale ticketing market may use decision heuristics, relying on past experiences and recognition-primed strategies [13,14].

Additionally, the different consumers of sports products are further divided into various segments of sports enthusiasts who possess a comprehensive understanding of the product elements and exhibit a significant psychological and emotional connection to it [15]. Passionate sports fans closely track daily game statistics, player salaries, and injury reports and have a profound emotional attachment to their team and players [16]. This creates a fanatic culture in which die-hard sports fans consistently follow and discuss the latest updates and real-time information through dedicated sports media channels and social media platforms online [17,18].

The considerable level of uncertainty in sports events creates anxiety and suspense among sports fans, which can lead to irrational and superstitious behavior [19,20]. This uncertainty also produces a wide range of opportunities for gambling and the legitimization of derivative markets, such as fantasy football [21,22]. Despite this uncertainty surrounding the sports environment, highly involved sports fans are convinced that they can better predict the outcome of sports events to some extent [23]. They tend to believe that they have more control over sporting events than they actually do and often believe they can influence the outcome [24].

The goal of this study is to explore how individual trait factors, such as experience, expertise, and fan involvement, affect the subjective accuracy of sports consumers’ inter-temporal purchase decisions. Understanding how these factors interact and influence decision-making under uncertainty can lead to a better understanding of consumer behavior in the advance ticket sales market.

## 2. Theoretical Background

### 2.1. Decision-Making under Uncertainty

Decision-making under uncertainty is a complex process that has been studied in various fields of behavioral science [2,14,25]. From the perspective of normative economics, it involves individuals making choices with incomplete information about the chances of potential outcomes [14]. Essentially, making decisions under uncertainty requires evaluating risks to quantify the level of uncertainty [12]. Effective decision-making requires more than just familiarity with factual information, concepts, and relationships. It also requires metaknowledge, which is an awareness of the limits of our knowledge [26].

Unfortunately, humans have a tendency to be overly confident in their beliefs and judgments, which can make decision-making under uncertainty more challenging [27]. Because meta-knowledge is not recognized or rewarded in practice (it is difficult for consumers to evaluate whether their purchase decision is relatively accurate), overconfidence has remained a hidden flaw in decision-making [28]. Bounded rationality is a theory that explains why consumers may struggle to make optimal decisions under uncertainty, as it suggests that individuals make decisions based on their cognitive abilities and the information available to them [29].

Consequently, decision-makers are frequently swayed by biases and heuristics that result in suboptimal decisions. For instance, when purchasing pre-sale tickets, consumers may adopt a straightforward dominant decision-making rule where one factor dominates their thought process. This could involve prioritizing the risk of the tickets selling out over finding the best deal on the price or vice versa [20]. Alternatively naturalistic decision-making suggests that experts use recognition-primed strategies to make decisions under uncertain circumstances by recognizing situations as prototypes stored in their long-term memory [13]. In the advance sales ticket market, experienced buyers should roughly know what to expect, leading to heuristic decision-making where consumers rely on past experiences to guide their current decisions [30]. This approach is particularly relevant in complex and dynamic environments, such as the pre-sale ticketing market for sports events, where there is a high degree of uncertainty and rapid changes in demand and prices [31].

### 2.2. Fan Involvement and Risk Perceptions

Any time customers consider purchasing a new product or service, they face a set of uncertainties about the product or service, which is collectively referred to as perceived risk [32,33]. Perceived risk consequentially determines whether the purchase is worth making or not [34]. In the context of sports, this includes general risks associated with attending games, such as the risk of injury, violence, or other negative events [35]. Studies on sport risk management indicate that the degree of fan involvement with their preferred sports team can impact their risk perceptions [34,35,36]. Research has found that high levels of fan involvement generally led to lower risk perceptions and higher levels of risk-taking behavior [37]. This is frequently attributed to the emotional attachment that fans have with their teams, which may cause them to perceive the risks associated with attending games as lower than they actually are [25]. In addition to emotional attachment, the level of control that fans perceive they have over the situation can also influence their risk perceptions [24]. This is due to the “illusion of control” bias, where individuals tend to overestimate their control over events that are actually determined by chance [38].

In the context of purchasing sports event tickets online, Dwyer et al. introduced the generic advanced decision model, which posits that the purchase decision is primarily influenced by two types of perceived risks: expected ticket availability (ETA) and expected likelihood of finding a lower rate (ELR) [39]. Those with higher a ETA are more inclined to book their tickets early, while those with a lower ELR are less likely to book their tickets right away. The authors’ empirical research also revealed that, on average, avid sports fans showed significantly lower expected ticket availability and a higher expected lower rate than the average group of consumers [39].

The degree of risk that fans associate with attending sporting events can be influenced by their level of involvement with their favorite team [34]. Fans who are emotionally invested may perceive the risks of missing out on their favorite event to be higher than the average consumer [25]. This perception can also be shaped by the amount of control that fans feel they have over the situation. Fans who believe they have more control over the ticket purchase process may underestimate the risks of sellout. Furthermore, fans who are confident in their ability to find the best deals may delay their purchase and continue searching for better deals in the future. However, these boundary conditions have yet to be examined in the sport literature.

Accordingly, the following hypotheses were developed and tested in this study:

**Hypothesis 1** **(H1).** *Individuals’ level of fan involvement will have a positive effect on their expected ticket availability (ETA).*

**Hypothesis 2** **(H2).** *Individuals’ level of fan involvement will have a positive effect on their expected likelihood of fining a lower rate (ELR).*

### 2.3. Illusion of Control and Sports

The illusion of control is a cognitive bias that refers to the tendency of individuals to overestimate their ability to control events that are actually determined by chance or other external factors [40]. This phenomenon has been extensively studied in the context of gambling, where players often believe that their actions can influence the outcome of a game of chance, such as rolling a dice or spinning a roulette wheel [38,41]. Empirical research has provided support for the existence of the illusion of control across a variety of domains, including sports [42], health [43], and business [44]. Studies have also shown that the illusion of control can have both positive and negative consequences. On the one hand, it can lead to increased motivation and persistence in pursuing goals [45,46]. On the other hand, it can lead to overconfidence and poor decision-making, especially in situations where chance plays a significant role [47,48].

One area where the illusion of control has been studied in sports is in relation to superstitious behaviors [49]. Superstitions are irrational beliefs or practices that are believed to bring about a desired outcome. For example, athletes may wear lucky socks or engage in specific rituals before a game [50]. Research has shown that athletes who engage in superstitious behaviors tend to have a greater sense of control over their performance, even though the behavior itself is not directly related to their performance [19]. In other words, athletes who engage in superstitious behaviors may believe that they have more control over the outcome of the game than they actually do [51].

The illusion of control has also been shown to influence the decision-making of athletes. Due to the overestimation of their control over events, athletes may make choices that involve greater risk or rely on incomplete information to make decisions. This can lead to negative outcomes since they may not have the level of control they believed they had. Studies conducted on this topic have shown that athletes may choose to engage in riskier behaviors or make decisions based on incomplete information because they believe they have more control over the outcome than they do [52].

Studies have also examined the illusion of control in relation to the perception of fairness in officiating in sports. It has been found that both athletes and fans tend to believe that they have more control over the outcome of the game when the officiating is perceived to be fair [53]. This belief can lead to a greater sense of control over the outcome of the game, even though the actual outcome is determined by the performance of the athletes [54]. Research in this area highlights how the illusion of control can impact the perception of fairness and influence the decision-making processes of athletes and fans.

### 2.4. Fan Involvement and Overconfidence

As previously mentioned, the illusion of control can significantly affect individuals’ behavior and decision-making in sports, particularly through the overconfidence bias of sports fans [23,24]. Fan involvement is a term used to describe the extent of emotional attachment and identification an individual has with a sports team or event [55]. The greater the emotional investment, the more likely the individual is to perceive a lower risk associated with attending games, despite the actual risks present.

This emotional attachment may cause fans to be blinded to the real risks, leading to overconfidence in their decision-making. Fans who have a strong attachment to a particular team or sport tend to believe they have superior knowledge and understanding of the game compared to others. This overconfidence among avid sports fans can result in various actions, such as overly optimistic predictions about game outcomes, underestimating the abilities of rival teams or players, and overestimating their capacity to affect the result of a game [56,57].

Accordingly, the following hypothesis was developed to test for the mediation effect of overconfidence:

**Hypothesis 3** **(H3).** *The relationship between involvement and risk perceptions (ETA and ELR) will be moderated by the individual’s level of confidence, acting as a mediator.*

**Figure 1:**
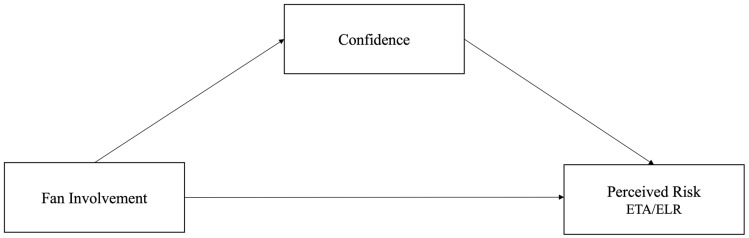
Hypothesized mediation model of fan involvement and confidence on risk perceptions.

The conceptual model in Figure 1 depicts the hypothesized mediation model relationships between the independent and dependent variables of the study:

To sum up, decision-making under uncertainty is a multifaceted and intricate process that is affected by various factors, such as risk probability calculations, past experiences, and cognitive heuristics. Understanding the mechanisms that shape consumer decision-making can provide valuable insights for sports marketers and ticket distributors looking to impact consumer behavior in the sports industry. This study focuses on understanding how the decisional factors (fan involvement, confidence, experience, and expertise) impact fans’ booking behavior in the advance ticket sales environment. This understanding can also aid managers in developing effective strategies for ticket sales and marketing, as well as identifying potential areas of improvement in their sales process. Ultimately, a deeper understanding of fan behavior in the advance ticket sales environment can help sports organizations improve fan satisfaction and loyalty and ultimately drive revenue growth.

## 3. Methods and Measurements

### 3.1. Study Sample Demographics

To examine and test the effects of fan involvement and risk estimates of the advanced ticket sales environment, 640 respondents from Qualtrics were recruited to participate in the study. Ethical review and approval were not required for the study on human participants in accordance with the local legislation and institutional requirements. Through Qualtrics, the online survey was distributed to geographically confined subjects of New York City residents over a 10-day period from 7 December to 17 December 2017. Within each email, a brief message and link were provided to ask the respondents to participate in filling out an online questionnaire. Table 1 provides key demographic information for the sample.

**Figure 2:**
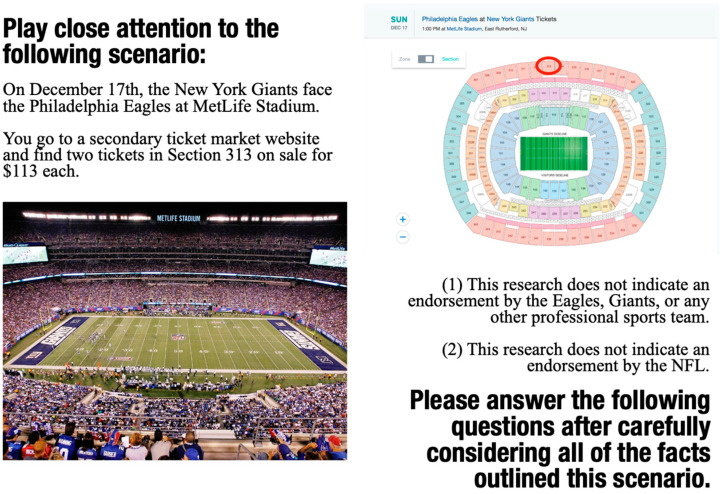
Vignette of the ticket purchase scenario.

The study adapted ELR and ETA measurements from the studies of Chen and Schwarz and Dwyer, and Drayer, and Shapiro’s quasi-experimental design on examining the booking behavior for sporting events [39,58]. The research subjects who agreed to participate in the study were given a vignette scenario, where a group of friends decides to watch a NY Giants home NFL game against the Philadelphia Eagles (view Figure 2).

### 3.2. Study Design and Procedures

The participants were told that they had been asked by their group of friends to book game tickets for the entire group. They searched the Internet to find available tickets to attend the future game. The described experimental task controlled for extraneous variables including the opponent for the game, the seat location, the date of stay, and the quoted price. After reading through the purchase scenario, the participants were asked two questions regarding estimating the likelihood of future events (the likelihood of sellout and the likelihood of finding a better priced deal later). The survey also collected the participants’ demographic information, followed by questions regarding their ticket purchase behavior, knowledge of sport ticket markets, and fan involvement.

The measurement methods used to operationalize the illusion of control have varied in the literature on consumer behavior and sports management [23,24]. Studies have used different measures, such as discrepancy scores between expected and actual performance, self-rated perceived control, and self-rated confidence. Presson and Benassi found that studies which measured participants’ perceived ability to predict outcomes had larger effect sizes than those that measured the perceived ability to control outcomes [59]. As such, this study will utilize self-rated perceptions of the likelihood of expected ticket availability (ETA) and the expected likelihood of finding a lower rate (ELR). The study also used single-item measures to directly assess the participants’ perceived probability of sellout risk and the likelihood of finding a better deal, adapted from Dwyer, Drayer, and Shapiro’s study on secondary ticket markets [39]. Demographic information, ticket purchase behavior, knowledge of sport ticket markets, and fan involvement will also be measured and collected.

### 3.3. Dependent Variables

A summary of all of the variables and survey measurement items are included in Table 2.

The two dependent variables for this study were the ETA, the subjects’ assessment of the expected ticket availability, and the ELR, their assessment of the likelihood of finding a similar or lower price ticket any time before the scenario date and the game.

#### 3.3.1. Expected Ticket Availability (ETA)

I believe the chance the same or very similar tickets will be available between tomorrow (DATE) and today (DATE) is _______%. (Please indicate a number between 0 and 100).

#### 3.3.2. Expected Likelihood of Finding a Better Rate (ELR)

I believe the chance that I could find the same or very similar tickets somewhere else at a price lower than USD (PRICE) each between tomorrow (DATE) and today (DATE) is ______%. (Please indicate a number between 0 and 100).

### 3.4. Independent Variable

#### Fan Involvement (Behavioral Involvement)

The following items were included to measure fan involvement: (1) the total number of years as a spectator and participant in the NFL; (2) the total number of hours spent watching and following their favorite NFL players and teams on a weekly basis; and (3) the amount of money they spend on NFL merchandise on a weekly basis. A weighted composite score of involvement was created through exploratory factor analysis (EFA).

### 3.5. Mediator Variable

#### Perceived Confidence

Three items were used to measure the participants’ perceived confidence (attitude) about their ticket purchase (“I am confident in using both primary and secondary ticket market knowledge compared to the average sport fan”, “I am confident in searching for the best deal available compared to the average sport fan”, and “Generally, I am confident in my purchase decision for sport games”). A seven-point Likert scale (1 = strongly disagree; 7 = strongly agree) was used to measure the variability among the three items.

### 3.6. Control Variables

To better examine the hypothesized relationships and the proposed mediation model, two potential covariates were introduced to the statistical control variance between the main and indirect effects of the proposed structural model.

#### 3.6.1. Ticket Purchase Experience

Two items were used to measure an individual’s past ticket purchase behavior. The first item asked how often the participants purchase tickets online, monthly, on a five-point scale (1 = never; 2 = rarely; 3 = sometimes; 4 = frequently; 5 = every month). The second item asked the participants to identify the number of days since they had last purchased a ticket online for a sports game. The measurement was reverse coded and deducted from a total value of 365 days to ensure the directional strength of measurement; 365 indicates that the consumer purchased a ticket today and 1 indicates that the most recent day when the consumer purchased a ticket is 1 year ago.

#### 3.6.2. Perceived Expertise of the Sports Ticket Market

Two items were adapted from Kwak et al. [24] to measure the participants’ perceived knowledge about sport ticket markets: “I am knowledgeable about sport ticket markets compared to the average sport fan”, “I have better ability to comprehend necessary sport information compared to the average sport fan”, and “I have better ability to comprehend sport ticket market information compared with the average sport fan”. The measures utilized a seven-point Likert scale (1 = strongly disagree; 7 = strongly agree).

## 4. Analysis

All statistical analyses were performed in R 3.6.2 and SPSS 27. Table 3 shows the number of observations that correspond to the 10-time level treatments as well as the averages and standard deviations for each of the two dependent variables (ELR and ETA). Perceived ticket availability was highest 10 days before the event (at 53.72%) and lowest the day before the event (at 37.89%). The same pattern was observed for the ELR. The ELR was highest at 48.92% 10 days before the event and dropped significantly to 35.88% just a day before the event. In general, as the time grew closer to the event, the respondents’ perceived probability of both ticket availability and the likelihood of finding a lower priced ticket dropped significantly.

Using Wilks’ statistic, our MANOVA results show that there was a significant effect of the time period on the participants’ ETA and ELR risk assessments [Λ = 0.954, *F*(18, 1262) = 1.653, *p* < 0.05]. The descriptive statistics for the different dependent variables are reported in Table 4. The effect size measured using partial eta-squared values η^2^ was 0.023. Because the participants responded over 10 different time slots for the experiment, time also needed to be included as a control variable for the global mediation model. Therefore, a time dummy variable was generated based on when the respondent took the survey prior to the days leading to the game event scenario.

Before conducting regressions for hypotheses testing, weighted sum scores for involvement, confidence, expertise, and experience were created through a series of exploratory factor analyses [60]. Next, a series of ordinary least squares (OLS) regression models were conducted to test the study’s hypotheses regarding the effects of the consumers’ level of involvement on their risk perception through the level of overconfidence. To isolate the effect of the independent variable and mediator, past experience, expertise was included as a control variable in the mediation model. Involvement and confidence predicted ETA, R^2^ = 0.063 and ELR, R^2^ = 0.94. Detailed regression estimates of the coefficients are presented in Table 4.

## 5. Results

**Figure 3:**
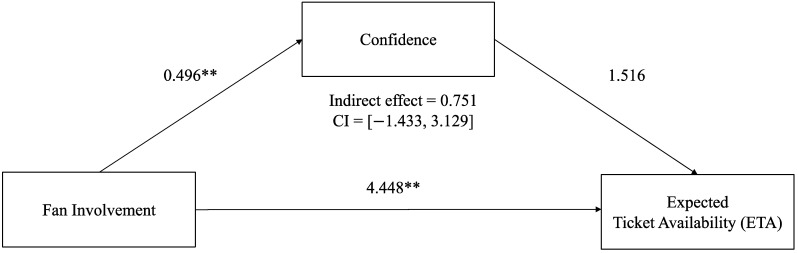
Unstandardized regression coefficients for the relationship between involvement and expected ticket availability (ETA) mediated by perceived confidence, ** *p* < 0.01.

**Figure 4:**
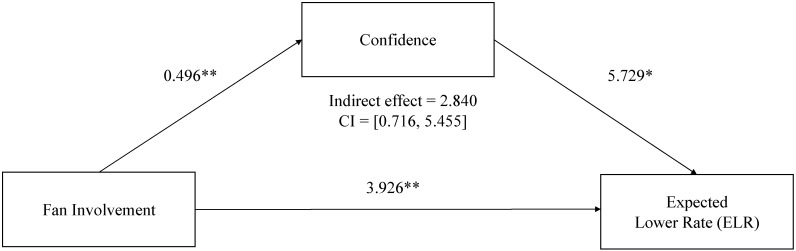
Unstandardized regression coefficients for the relationship between involvement and expected lower rate (ELR) mediated by perceived confidence, * *p* < 0.05, ** *p* < 0.01.

H1 was supported as fan involvement was a significant predictor of the level of confidence (*B* = 0.496, *p* < 0.001). Confidence was a significant predictor of the ELR (*B* = 5.729, *p* < 0.05). However, confidence was not significantly associated with the ETA *(B* = 1.516, *p* = 0.504). To examine the mediated relationship of the illusion of control (involvement, overconfidence, and probability judgments of risk perception), the study generated confidence intervals through bootstrapping methods [61]. The indirect effect of involvement on the ELR through overconfidence was found to retain 95% confidence intervals (CIs) that did not include zero (indirect effect = 2.840, CI = [0.716, 5.455]). However, the indirect effect of involvement on the ETA through overconfidence was not statistically significant as the 95% confidence interval includes zero (indirect effect = 0.751, CI = [−1.433, 3.129]). Thus, H3 was partially confirmed (only for the ELR and not significant for the ETA). Figure 3 and Figure 4 display the hypothesized path model results including the unstandardized regression coefficients and confidence intervals through bootstrapping methods.

## 6. Discussion

The aim of this research was to investigate how individual trait factors, such as experience, expertise, and fan involvement, impact the subjective accuracy of sports consumers’ intertemporal purchase decisions. The study hypothesized that fan involvement would lead to overconfidence bias in risk perceptions for sport fans in the presale ticketing environment, based on the illusion of control theory. The findings revealed that high involvement did, in fact, result in an overestimation of risk perceptions (ETA and ELR) in the presale ticketing environment, and this relationship was partly mediated by consumers’ overconfidence.

### 6.1. Theoretical Implications

Dwyer, Drayer, and Shapiro first found that provided with the same environmental ticket market scenario, the average ETA (47.7% > 40.7%) and ELR (44.8% > 36.6%) predictions were higher for high involvement fans than low involvement fan groups [39]. These results indicate that the general prediction for availability and the perceived likelihood of finding a better deal were generally higher for highly identified sports fans [39]. Although this significant difference across fan groups has been an intriguing empirical finding, there has not been any research on the root cause of and procedural decision-making explaining this effect.

One key factor that this study considers as a root cause is the overconfidence that is created by consumer involvement in their product [36]. This study focuses on the concept of overconfidence that arises due to consumers’ emotional involvement with the product. It uses the illusion of control theoretical framework to investigate why highly involved sports fans can be blinded by overconfidence in their decision-making, leading to biases in future probability judgments and inter-temporal choices. The study tests whether varying levels of fandom affect the risk perception of future ticket availability and the likelihood of finding a better deal in the same environmental ticket market purchase conditions.

One explanation for the relationship between fan involvement and the ETA is that highly involved fans may be more likely to engage in selective attention and biased processing of information related to their team or sport [62]. This can lead to a confirmation bias, where fans selectively attend to and interpret information that supports their pre-existing beliefs and ignore information that contradicts them [63]. For example, a fan who is highly involved with a particular team may only pay attention to statistics or news stories that support their belief that their team will be likely to sell out, while ignoring or dismissing information that suggests otherwise [53].

Alternatively, another possible explanation for the relationship between fan involvement and the ELR is that highly involved fans may feel a greater sense of control over the outcome of a game [64]. This sense of control can lead to an illusion of control, where fans overestimate their ability to influence the outcome of a game through their own actions or support of their team. For example, a highly involved fan may believe that wearing a particular jersey or performing a certain ritual before a game will increase their team’s chances of winning [49].

Overall, we theorized that customers may be blinded by their attachment, which clouds there judgement and risk perception judgment towards the uncertain purchase environment. In addition, this overconfidence would in fact be exhibited through overestimation of their ability to control events and, relatedly, the overestimation of their task skill ability to secure lower priced deals for a given ticket. The mediation analysis in our study was run as a confirmatory assessment of the overall theory of illusion of control being posited in this empirical study. The apparent mediation effect of overconfidence between involvement and the ELR in subjective risk probability in this study hopes to further strengthen the idea that given the same environmental scenario of uncertainty, consumers do not evaluate the information cues equally and objectively.

### 6.2. Practical Implications

The results of this study have several important implications for sports marketing and management, as they can help managers better understand how fans perceive and engage with their teams and events. For example, if managers can identify the factors that influence fans’ booking behavior, they can tailor their marketing strategies to better meet the needs and preferences of their target audience [62].

First, the findings suggest that fan involvement is a crucial factor to consider when predicting consumer behavior in the sports industry [15]. Fans who are more involved with a particular sport or team are more likely to believe that they will be able to obtain tickets for a game and are also more likely to believe that they will be able to find a better deal on those tickets. This finding suggests that sports marketers should focus on building strong relationships with fans and creating a sense of community around their teams in order to increase fan involvement and engagement.

Second, the results of this study suggest that sports marketers should pay attention to the timing of ticket sales. Specifically, the study found that fans are more likely to believe that tickets will be available and that they will be able to find a better deal on those tickets as the event date approaches. This empirical finding suggests that sports marketers should consider implementing dynamic pricing strategies that take into account the expected demand for tickets as the event date approaches, as well as promotional strategies that incentivize fans to purchase tickets earlier rather than later.

Third, the study suggests that sports managers and marketers should focus on building consumer confidence in the ticket purchasing process. The study found that fan confidence is positively associated with expectations of ticket availability, which in turn influences purchase decisions. Although overconfidence might distort the prediction accuracy of agents in decision-making, it can still serve a purpose during the decision implementation process (i.e., higher confidence in the decision can lead to higher satisfaction when decisional efficiency cannot be attained). This finding suggests that sports marketers should implement strategies that build consumer confidence in the ticket purchasing process, such as offering clear and transparent pricing information and providing easy-to-use online ticket purchasing platforms.

Overall, the findings of this study provide valuable insights into the factors that influence consumer behavior in the sports industry. By understanding how fans perceive risks and make decisions, managers can optimize the pricing and availability of tickets to maximize revenues while still providing a positive experience for fans. Ultimately, a deeper understanding of the factors that influence decision-making in sports can help managers improve their business operations and enhance the overall fan experience.

### 6.3. Limitations and Future Research

Although this study might offer interesting implications towards practice, several limitations should be noted. Firstly, the study was conducted using an online survey that used self-reported measures, which may be subject to response bias and not capture the full range of responses and behaviors that may occur in a real-life ticket purchase scenario [65]. Secondly, a particular team brand (the NY Giants and the Philadelphia Eagles) was used to control for the potential effects of brand strength. Although Gierl et al. [66] did not find any significant difference between a famous brand and fictitious generic brands in their quasi-experimental study, the potential moderating effects of brands might be worth examining in this area of research. Thirdly, the study focused only on the impact of fan involvement and perceived risk on ticket purchase decisions, and other factors such as price sensitivity and social influence were not explored [67]. Lastly, the study did not find a significant mediating effect between the relationship of involvement and ticket availability. We posit alternative cognitive biases to better explain why fan involvement may influence scarcity perceptions [68].

Despite these limitations, the study provides valuable insights into the inter-temporal decision-making process of sports fans when purchasing tickets for sporting events. Future research can expand on these findings by exploring additional factors that influence ticket purchase decisions and by examining other contexts such as on-site ticket purchases and secondary ticket markets. Additionally, future research can investigate the impact of these factors on other consumer behaviors, such as concession sales and merchandise purchases, in the sports industry.

## 7. Conclusions

In conclusion, decision-making in the context of purchasing tickets for sporting events is a complex and multifaceted process influenced by several factors, including the perception of risk, fan involvement, confidence, experience, and expertise. The advance ticket sales market for sporting events presents a challenging environment for consumers to make accurate purchase decisions due to the significant level of uncertainty surrounding the sports product. Fans’ decision-making processes can be distorted by cognitive biases and heuristics, leading to suboptimal choices. The study has replicated the illusion of control and revealed that overconfidence bias exists in the pre-sale ticketing environment. Understanding the factors that influence fans’ decision-making processes can help sports marketers and ticket distributors better understand how fans engage with their teams and events, and ultimately improve their sales and marketing strategies. Further research is needed to explore these relationships in more depth and to develop interventions to help fans make more informed decisions.

## Figures and Tables

**Table 1:** Key demographics of the sample.

Age	33.674, Mean	Ethnicity	76.3%, Caucasian
	11.792, St. Dev		12.3%, Hispanic
Gender	69.1%, Male		6.1%, Asian
	30.9%, Female		5.3%, Other
	12.5%, Less than USD 20 K		21.5%, High school
Household Income	20.7%, USD 20 K–USD 40 K	Education	4.1%, Bachelor’s degree
	22.9%, USD 40 K–USD 60 K		18.0%, Graduate degree
	24.7%, USD 60 K–USD 80 K		16.5%, Professional degree
	15.0%, USD 100 K–USD 200 K		7.7%, Other
	3.4%, More than USD 200 K		9.0%, Did not specify

**Table 2:** Summary of the measurement items.

Variables of Study	Survey Items
Dependent Variable	
ETA	I believe the chance the same or very similar price tickets will be available between tomorrow and today is ______ %
ELR	I believe the chance I can find the same or very similar price ticket deals between tomorrow and today is ______ %
Independent Variable	
Involvement	Total number of years as a spectator or participant in the NFL
	Total number of hours spent watching or following their favorite NFL players or teams per week
	Total amount of money spent on NFL merchandise per year
Mediator Variable	
Confidence	I am confident in using both primary and secondary ticket market knowledge compared to the average sport fan
	I am confident in searching for the best deal available compared to the average sport fan
	Generally, I am confident in my purchase decisions for sports games
Control Variables	
Expertise	I am knowledgeable about sport ticket markets compared to the average sports fan
	I have a better ability to comprehend necessary sport ticket market information compared to the average sports fan
Experience	How often do you purchase tickets online on a monthly basis?
	Number of days since you last purchased a ticket online for the NFL
Time	Number of days before the game of event (dummy coded)

**Table 3:** Estimates of the ETA and the ELR by days leading up to the event.

		Expected Ticket Availability		Expected Lower Rate	
Days before the Game	Number of Observations	Average (%)	SD	Average (%)	SD
10	40	53.72	28.88	48.92	29.19
9	80	52.13	24.09	48.13	23.57
8	54	50.33	25.85	46.22 *	28.49
7	48	46.22 *	28.47	44.93	28.83
6	71	47.36 **	28.95	42.53 *	24.32
5	80	50.08	29.97	43.98	27.65
4	56	48.73	29.81	44.94	28.42
3	60	40.33 *	22.68	44.31 *	24.18
2	76	40.81 ***	27.98	38.63 ***	29.21
1	76	37.89 ***	28.15	35.88 **	29.75

* *p* < 0.05, ** *p* < 0.01, *** *p* < 0.001.

**Table 4:** Regression estimates of unstandardized coefficients.

	Confidence		Expected Ticket Availability		Expected Lower Rate	
	Estimate	SE	Estimate	SE	Estimate	SE
Involvement	0.496 ***	0.069				
Involvement			4.448 **	1.639	3.926 *	1.655
Confidence			1.516	2.269	5.729 *	2.25
D1			19.881 ***	5.695	18.067 **	5.503
D2			16.273 ***	4.308	16.041 ***	4.503
D3			12.389 *	4.925	10.419 *	5.254
D4			8.371	5.169	9.530	5.192
D5			9.075	4.717	6.130	4.438
D6			13.925 **	4.709	9.202 *	4.591
D7			11.489 *	4.986	9.702	5.022
D8			3.180	4.345	8.305	4.611
D9			8.279	4.832	10.130 *	4.948
Expertise			0.625	2.207	−2.488	2.316
Experience			2.264	1.669	2.877	1.715

Total R^2^ = 6.3% (ETA), 9.4% (ELR), *N* = 641. * *p* < 0.05, ** *p* < 0.01, and *** *p* < 0.001.

## Data Availability

Raw data supporting the conclusions of this article will be made available by the authors, without undue reservation.
